# Supplementary material for: Orientia tsutsugamushi Infection in Wild Small Mammals in Western Yunnan Province, China
Source: Pathogens. 2023 Jan 12;12(1):128. doi: 10.3390/pathogens12010128 (PMC9862787; doi:10.3390/pathogens12010128)
Supplement: Supplementary file 1 [file pathogens-12-00128-s001.zip › pathogens-2063252-supplementary.pdf]

Table S1. *Ot* strains from Genbank used in this study

Species of strain	Source	No. Genbank	Geographic location	Time	DNA size(bp)
Karp	Human	M33004	New Guinea	1943	1599
Taiwan Gilliam	–	DQ485289	China Taiwan	2006	1860
Kuioki	Human	M63380	Japan	1981	1599
KM11-2	Human	GU446591	China Taiwan	2010	1608
KG_ST27	Human	MG870317	India	2018	558
NAN057	Human	MK660515	Thailand	2019	468
OtMMC73	–	MK617204	Bangladesh	2018	721
OTVi18	Human	KY635436	India	2017	522
Sxh951	Human	AF050669	China	1998	1544
OTSg62	Human	KF777346	India	2013	315
DS3910C	Chiggers	MK660524	Thailand	2019	495
Boryong	Human	L04956	South Korea	1993	1874
OTE7	Human	KY635435	India	2017	627
KM21-1	Chiggers	GU446605	China Taiwan	2010	1575
CM606	Human	HM777460	Thailand	2010	550
DS2641C	Chiggers	MK660519	Thailand	2019	477
TT0711a	Human	GQ332755	China Taiwan	2009	1551
YNHH65	*Eothenomys miletus*	KU664516	China	2016	151

Figure S1. Phylogenetic tree based on *Ot* 56kDa gene fragment constructed by a maximum likelihood method.

Notes: ● Positive Samples of Lianghe; ■ Positive Samples of Jianchuan; ▲ Positive Samples of Yulong.

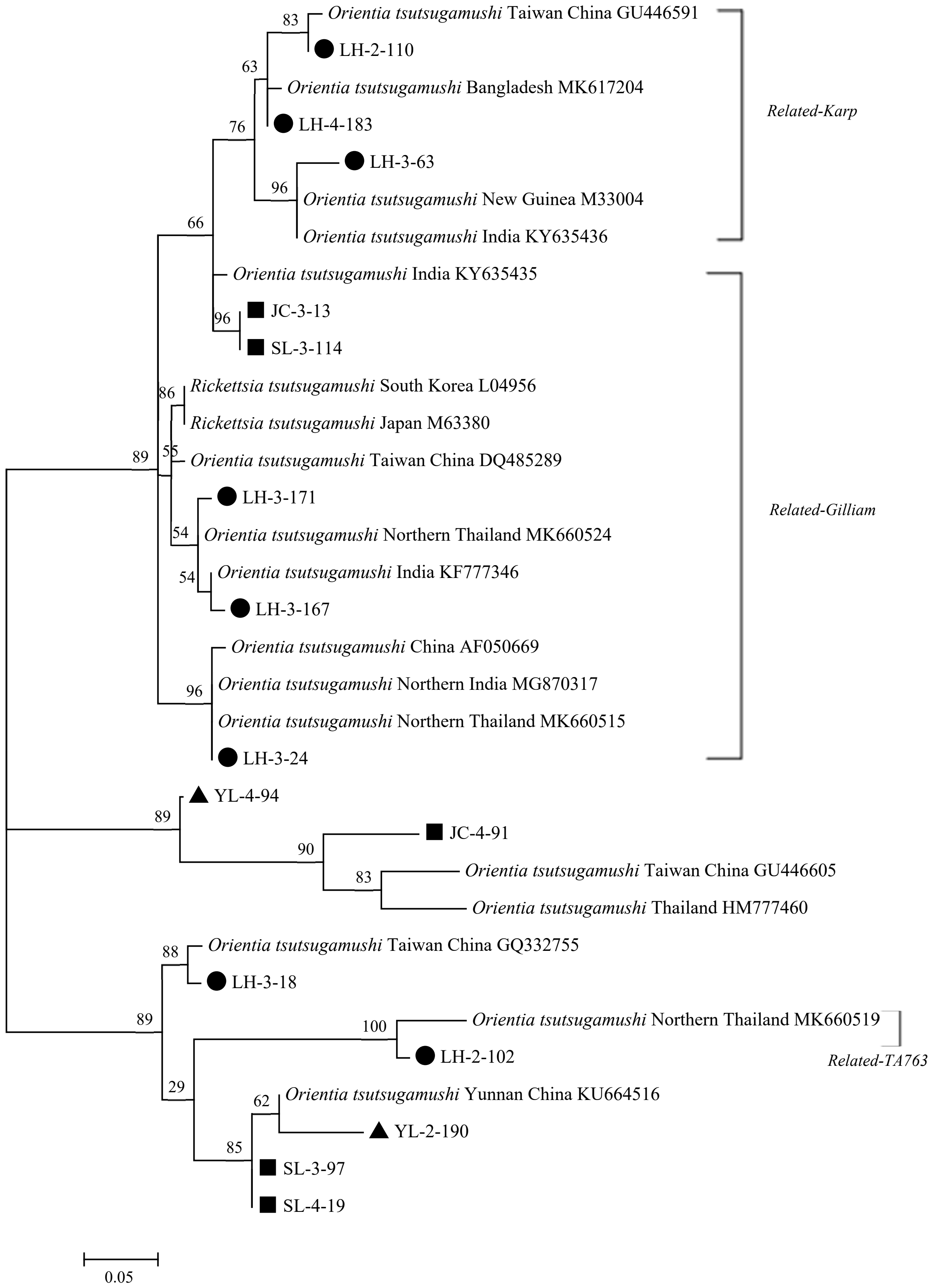

Table S2. *Ot* genotypes of the infected wild small mammals in western Yunnan Province

County	Small Mammals	Positive Sample*	Genotype
Lianghe	*Rattus sladeni*	LH-2-89	*related-Gilliam*
Lianghe	*Hylomys suillus*	LH-2-102	*related-TA763*
Lianghe	*Rattus tanezumi*	LH-2-107	*related-Karp*
Lianghe	*Rattus tanezumi*	LH-2-110	*related-Karp*
Lianghe	*Rattus tanezumi*	LH-3-002	*related-Karp*
Lianghe	*Anourosorex squamipes*	LH-3-18	*closer Kawasaki*
Lianghe	*Rattus sladeni*	LH-3-24	*related-Gilliam*
Lianghe	*Rattus sladeni*	LH-3-63	*related-Karp*
Lianghe	*Rattus sladeni*	LH-3-100	*related-Gilliam*
Lianghe	*Rattus sladeni*	LH-3-102	*related-Gilliam*
Lianghe	*Rattus sladeni*	LH-3-108	*related-Gilliam*
Lianghe	*Suncus murinus*	LH-3-162	*closer Kawasaki*
Lianghe	*Rattus sladeni*	LH-3-167	*related-Gilliam*
Lianghe	*Rattus tanezumi*	LH-3-171	*related-Gilliam*
Lianghe	*Rattus sladeni*	LH-4-116	*related-Gilliam*
Lianghe	*Rattus sladeni*	LH-4-134	*related-Gilliam*
Lianghe	*Niviventer fulvescens*	LH-4-139	*related-Gilliam*
Lianghe	*Rattus tanezumi*	LH-4-183	*related-Karp*
Jianchuan	*Eothenomys miletus*	SL-1-34	*closer Kawasaki*
Jianchuan	*Crocidura attenuate*	SL-3-56	*closer Kawasaki*
Jianchuan	*Eothenomys miletus*	SL-3-97	*closer Kawasaki*
Jianchuan	*Eothenomys miletus*	SL-3-114	*related-Gilliam*
Jianchuan	*Eothenomys miletus*	SL-3-115	*closer Kawasaki*
Jianchuan	*Apodemus draco*	SL-3-132	*closer Karp*
Jianchuan	*Eothenomys miletus*	SL-4-006	*closer Karp*
Jianchuan	*Eothenomys miletus*	SL-4-009	*closer Kawasaki*
Jianchuan	*Eothenomys miletus*	SL-4-18	*closer Karp*
Jianchuan	*Eothenomys miletus*	SL-4-19	*closer Kawasaki*
Jianchuan	*Eothenomys miletus*	SL-4-26	*related-Gilliam*
Jianchuan	*Eothenomys miletus*	SL-4-47	*closer Kawasaki*

Table S2. *Ot* genotypes of the infected wild small mammals in western Yunnan Province (Continued).

County	Small Mammals	Positive Sample	Genotype
Jianchuan	*Eothenomys miletus*	SL-4-106	*closer Kawasaki*
Jianchuan	*Apodemus draco*	JC-1-74	*closer Kawasaki*
Jianchuan	*Apodemus chevrieri*	JC-1-106	*closer Kawasaki*
Jianchuan	*Eothenomys miletus*	JC-2-43	*related-Gilliam*
Jianchuan	*Eothenomys miletus*	JC-2-45	*related-Gilliam*
Jianchuan	*Eothenomys miletus*	JC-2-78	*closer Kawasaki*
Jianchuan	*Eothenomys miletus*	JC-3-13	*related-Gilliam*
Jianchuan	*Apodemus draco*	JC-3-55	*related-Gilliam*
Jianchuan	*Apodemus chevrieri*	JC-3-66	*closer Kawasaki*
Jianchuan	*Niviventer andersoni*	JC-4-91	*closer Karp*
Yulong	*Niviventer confucianus*	YL-2-190	*closer Kawasaki*
Yulong	*Apodemus draco*	YL-3-288	*closer Karp*
Yulong	*Apodemus draco*	YL-4-94	*closer Karp*
Yulong	*Apodemus draco*	YL-4-102	*closer Karp*
Yulong	*Apodemus latronum*	YL-4-121	*closer Karp*

*Notes: LH: Lianghe county; JC and SL: Jianchuan county; YL: Yulong county.

1: Spring; 2: Summer; 3: Autumn; 4: Winter.
